# Geometric correction method for 3d in-line X-ray phase contrast image reconstruction

**DOI:** 10.1186/1475-925X-13-105

**Published:** 2014-07-29

**Authors:** Geming Wu, Mingshu Wu, Linan Dong, Shuqian Luo

**Affiliations:** 1 School of Biomedical Engineering, Capital Medical University, Beijing, China

**Keywords:** Projection data correction, Tomography reconstruction, In-line X-ray phase contrast imaging

## Abstract

**Background:**

Mechanical system with imperfect or misalignment of X-ray phase contrast imaging (XPCI) components causes projection data misplaced, and thus result in the reconstructed slice images of computed tomography (CT) blurred or with edge artifacts. So the features of biological microstructures to be investigated are destroyed unexpectedly, and the spatial resolution of XPCI image is decreased. It makes data correction an essential pre-processing step for CT reconstruction of XPCI.

**Methods:**

To remove unexpected blurs and edge artifacts, a mathematics model for in-line XPCI is built by considering primary geometric parameters which include a rotation angle and a shift variant in this paper. Optimal geometric parameters are achieved by finding the solution of a maximization problem. And an iterative approach is employed to solve the maximization problem by using a two-step scheme which includes performing a composite geometric transformation and then following a linear regression process. After applying the geometric transformation with optimal parameters to projection data, standard filtered back-projection algorithm is used to reconstruct CT slice images.

**Results:**

Numerical experiments were carried out on both synthetic and real in-line XPCI datasets. Experimental results demonstrate that the proposed method improves CT image quality by removing both blurring and edge artifacts at the same time compared to existing correction methods.

**Conclusions:**

The method proposed in this paper provides an effective projection data correction scheme and significantly improves the image quality by removing both blurring and edge artifacts at the same time for in-line XPCI. It is easy to implement and can also be extended to other XPCI techniques.

## Background

In recent years, X-ray phase contrast imaging (XPCI) has attracted much attention in many areas, such as bio-medical imaging [1], material science [2] and paleontology [3]. Different from conventional absorption-based X-ray imaging, XPCI uses the information of wave front changes while X-ray radiation passes through a sample. It produces refraction-sensitive images and reveals the internal anatomy of soft tissues with differences in the refractive indices, which can be described in the complex form as follows

(1) n=1−δ+iβ

where δ is the decrement of the real part of the refractive index, and β presents the absorption index of local attenuation. It is proved that δ is three orders of magnitude larger than β within the diagnostic X-ray range for human tissue [4]. Thus XPCI can greatly enhance the contrast for soft tissues in biological sample with high spatial and temporal resolutions than conventional absorption-based X-ray imaging technique.

To reveal the morphology of a thick biological sample by using XPCI, 2D projection images are collected by titling the sample around a fixed axis, which is perpendicular to the beam direction, and computed tomography (CT) technique is used to obtain 3D visualization of the internal structure of the sample. Usually standard filtered back-projection algorithm (FBP) or algebraic reconstruction technique (ART) [5] are employed to build CT slice images from projections. However, collected projections cannot be used for CT reconstruction directly without pre-processing in practice. Due to mechanical system with imperfect or misalignment of X-ray source, rotary stagy and CCD detector, an unavoidable problem encountered before CT reconstruction is how to align 2D projections and make the rotation axis coincident with the center line of each projection. Similar problem has been investigated in electron tomography (ET). A conventional scheme for this issue in ET is to employ gold particles or image derived markers to correctly align projections by computing geometric parameters from tracking and measuring projected positions of them [6,7]. However, this method needs additional components to generate accurate and measurable markers in projection images and thus makes the imaging procedure and post processing complex. Some marker-free methods are also proposed to handle this issue in ET by using the cross-correlation (CC) of successive projection images [8] or taking specimen features as built-in markers for deriving geometric parameters [9,10]. These methods are based on pattern matching and have the drawback of accumulating alignment errors by comparing successive projection images. Recently, a mass center (MC) based method [11,12] is proposed to align 2D projection images before reconstructing 3D tomography image in coherent diffraction imaging (CDI) [13,14]. This method does not use local features but mass center as the invisible marker. Due to only one marker can be used, MC-based method works well for translational alignment, but poorly for rotational alignment. And it is sensitive to background noise and thus needs a well-designed background subtraction scheme. Some existing software suites developed for XPCI reconstruction usually provide a manual and automatic integrated approach to handle the data correction issue before CT reconstruction. PITRE [15] employs a MC-based sinogram correction scheme to calibrate the location of rotation axis, and the user can refine the result manually. DEIRecontructor [16] calculates geometric parameters by using user-selected markers. In this paper, a new automatic method is presented to solve the data correction problem in in-line XPCI, which is a propagation-based phase contrast imaging technique. This method employs an iterative approach to properly determine optimal geometric parameters. And a two-step method, which includes performing a composite geometric transformation and then following a linear regression process, is used to find out the solution at each iteration. Numerical experiments on synthetic and real image datasets demonstrate that the proposed method provides a fast and reliable image pre-processing scheme for CT reconstruction in in-line XPCI.

The rest parts of this article are organized as follows: Methods section describes the projection data misplacement problem for CT reconstruction in in-line XPCI and its mathematical model with primary geometric parameters at first, and then introduces the proposed method in details. Results section shows numerical experimental results from synthetic and real datasets which are pre-processed by the proposed method, and followed by the study conclusion in Consclusions section.

## Methods

### Projection data correction in in-line XPCI

**Figure 1:**
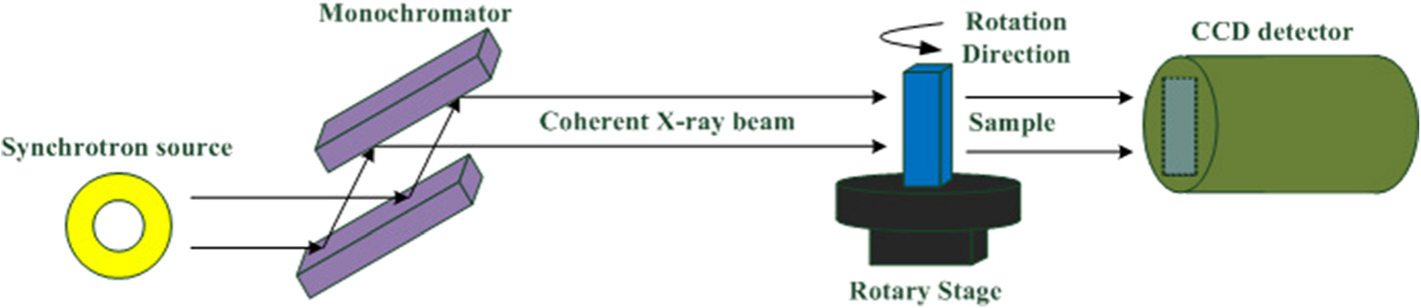
The schematic set-up of in-line XPCI.

XPCI uses a spatially coherent beam and an X-ray sensitive detector to acquire projection images from a sample. To obtain 3D structural information of the sample, a rotary stage with high precision is used to put the sample on and a series of projections collected while titling the sample around a rotation axis with respect to the base of stage. In-line XPCI [17] is a propagation-based phase contrast imaging technique and the most simple and straight-forward method to achieve X-ray phase contrast images. A synchrotron X-ray source, the sample and an X-ray sensitive detector are arranged in line as in conventional radiography but the detector is placed behind the sample in a certain distance. Figure 1 shows the schematic set-up of in-line XPCI.

While reconstructing slice images from acquired projections in in-line XPCI, the center line of any projection is considered to be identical to the rotation axis by default. Following this assumption, reconstruction algorithms, such as FBP, can be employed to build slice images correctly. However, it is usually very hard and time-consuming to align the components of imaging system with micrometer precision. If no data pre-processing step is adapted, the final reconstructed slice images will be blurred and with edge artifacts.

**Figure 2:**
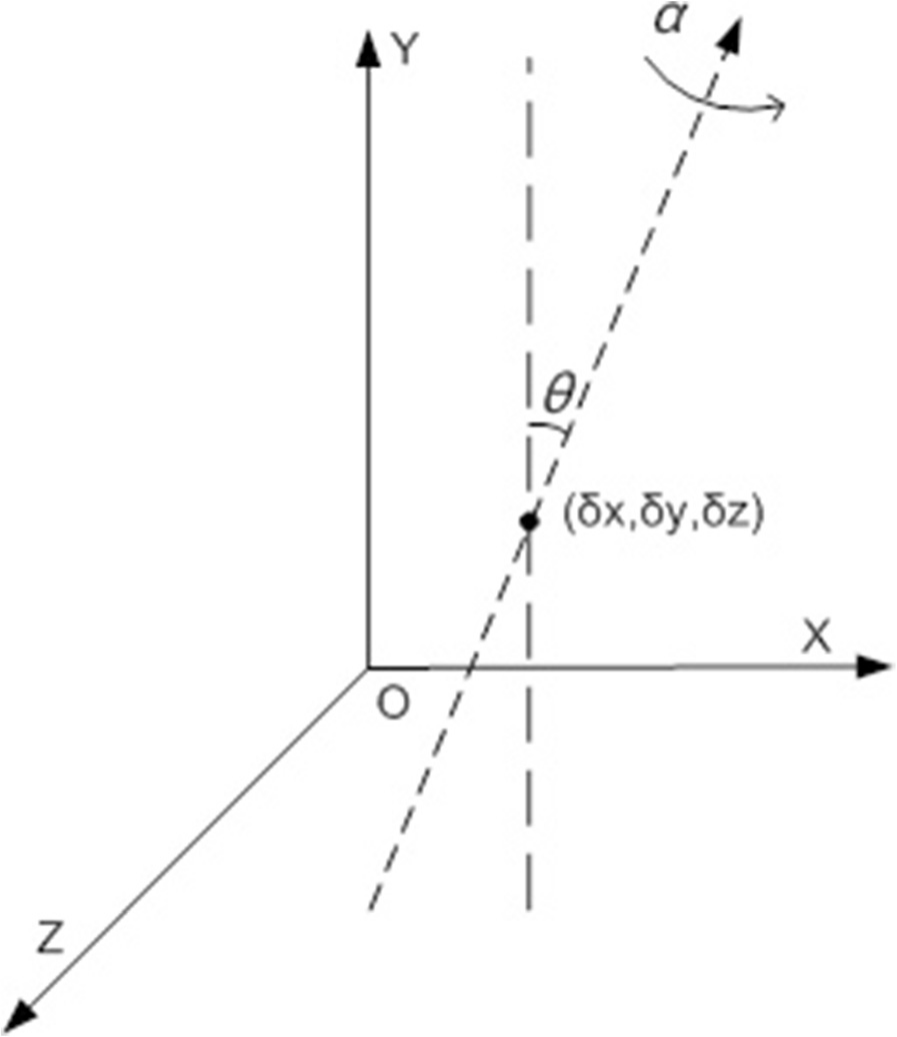
**Parameters in geometric model.***θ* denotes the in-plane flip angle and (*δ*_*x*_, *δ*_*y*_, *δ*_*z*_) are offsets along X, Y and Z directions respectively.

**Figure 3:**
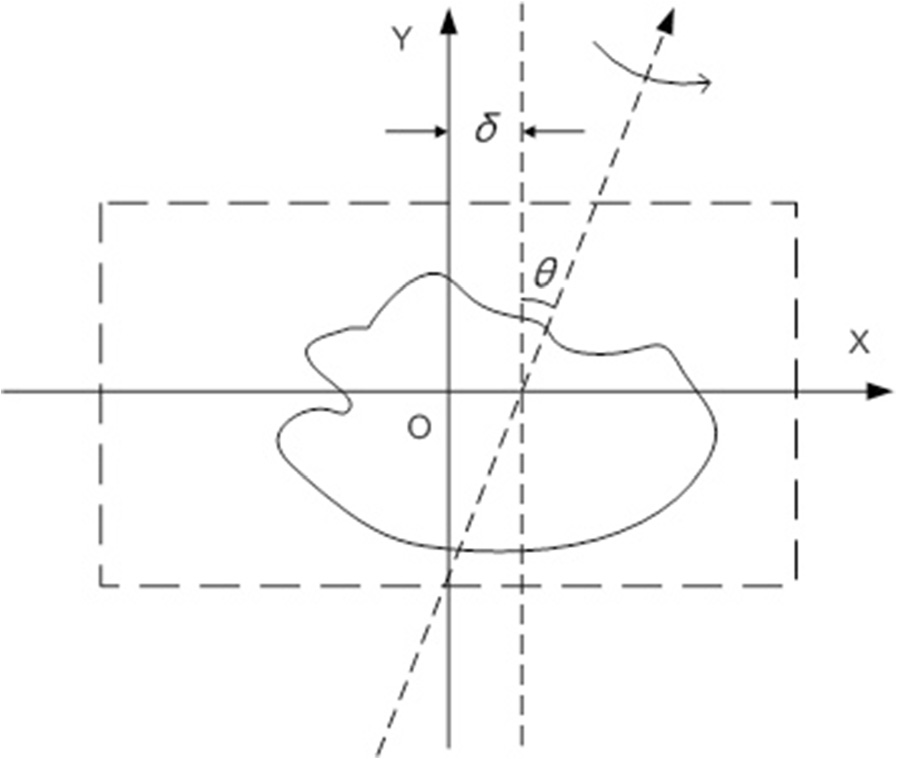
**Geometric parameters in simplified model.***θ* denotes the in-plane flip angle and *δ* is the offset from rotation axis to the center of projection image along X. Dashed box presents the projection window.

Suppose that Y is along the center line of CCD projection and Z is along the X-ray beam direction. Considering CCD detector and X-ray beam source are fixed during the whole imaging process, we use a model with four geometric parameters to describe the space relationship between the center line of projection and the rotation axis at tilt angle α as illustrated in Figure 2. Here θ is the in-plane flip angle which specifies the slope of the rotation axis related to Y, and (*δ*_*x*_, *δ*_*y*_, *δ*_*z*_) are offsets along X, Y and Z directions respectively. *δ*_*y*_ and *δ*_*z*_ are caused by the movement biases of rotary stage, and *θ* and *δ*_*x*_ are mainly due to the misalignment of CCD detector and rotary stage. The movement biases of rotary stage are controlled by the precision of mechanical design, and thus *δ*_*y*_ and *δ*_*z*_ are usually small and can be ignored. So *θ* and *δ*_*x*_ are two main factors to be considered and the model is simplified as illustrated in Figure 3. There *δ*_*x*_ is denoted as *δ*. The existence of two misalignment parameters results in geometric transforms of the projection images. The parameter *θ* is related to the rotation transform of projection images while *δ* to translational transform along X. So the misalignment problem can be solved by applying inverse geometric transform to the projection images if we know the values of these geometric parameters.

**Figure 4:**
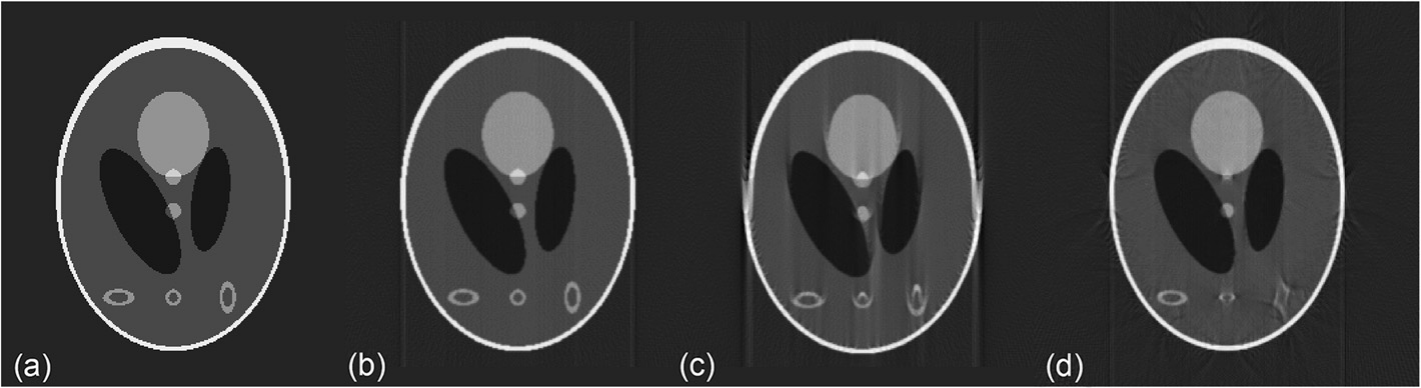
**Central slice reconstructed from projections of modified 3D Shepp-Logan phantom.** From left to right: **(a)** the original, **(b)** reconstructed without geometric transform, **(c)** reconstructed with *δ* = 2 pixels and **(d)** reconstructed with *θ* = -5*°*.

Figure 4 compares central slices reconstructed from projections of a modified 3D Shepp-Logan phantom [18] without and with geometric transforms by using FBP. Translational transform with *δ* = 2 pixels and rotation transform with *θ* = 5° are applied to projections respectively. Compared to the result without geometric transform, translational transform makes the slice image blurred while rotational transform introduces obviously edge artifacts.

### Two-step iterative correction method

In-line XPCI has no limitation on the range of tilt angle. Usually two projections with special tilt angle of 0 and 180 degree are included while collecting projections in in-line XPCI. Denote *P*_0_ and *P*_*π*_ the two projections with special tilt angles respectively. According to the physical principle of in-line XPCI, *P*_*π*_ is the reflection of *P*_0_ by taking the rotation axis as the mirror line. So *P*_*π*_ should be identical to the image which is achieved by applying the following composite geometric transformation to *P*_0_ in homogeneous coordinates

$$\begin{array}{ll} \left[\begin{array}{c} x^{\prime }\\ y^{\prime }\\ 1 \end{array}\right] & =\left[\begin{array}{ccc} 1 & 0 & \delta \\ 0 & 1 & 0\\ 0 & 0 & 1 \end{array}\right]\;\left[\begin{array}{ccc} \cos\theta & -\sin\theta & 0\\ \sin\theta & \cos\theta & 0\\ 0 & 0 & 1 \end{array}\right]\;\left[\begin{array}{ccc} -1 & 0 & 0\\ 0 & 1 & 0\\ 0 & 0 & 1 \end{array}\right]\;\left[\begin{array}{ccc} \cos\theta & \sin\theta & 0\\ -\sin\theta & \cos\theta & 0\\ 0 & 0 & 1 \end{array}\right]\;\left[\begin{array}{ccc} 1 & 0 & -\delta \\ 0 & 1 & 0\\ 0 & 0 & 1 \end{array}\right]\;\left[\begin{array}{c} x\\ y\\ 1 \end{array}\right]\\ & =\left[\begin{array}{ccc} \sin^{2}\theta -\cos^{2}\theta & -2\sin\theta \cos\theta & 2\delta \cos^{2}\theta \\ -2\sin\theta \cos\theta & \cos^{2}\theta -\sin^{2}\theta & 2\delta \sin\theta \cos\theta \\ 0 & 0 & 1 \end{array}\right]\;\left[\begin{array}{c} x\\ y\\ 1 \end{array}\right] \end{array}$$

where (*x*, *y*) is a point in *P*_0_ and (*x*′, *y*′) is its corresponding position in *P*_*π*_. If the rotation angle *θ* is small enough, then (*x*′, *y*′) is approximated by

(2) $$\left\{\begin{array}{c} x^{\prime }\approx -x+2\left(\delta -\mathrm{\theta y}\right)\\ y^{\prime }\approx y+2\left(\delta -x\right)\theta \end{array}\right)$$

Thus we obtain

(3) $$P_{0}\left(x,y\right)=P_{\pi }\left(2\left(\delta -\mathrm{\theta y}\right)-x,y+2\left(\delta -x\right)\theta \right)$$

The above equation shows that the optimal geometric parameters can be achieved by solving the following minimization problem when *P*_0_ and *P*_*π*_ are available

(4) $$\left(\theta^{*},\delta^{*}\right)=\arg\min_{\theta ,\delta }\iint {\left(P_{0}\left(x,y\right)-P_{\pi }\left(2\left(\delta -\mathrm{\theta y}\right)-x,y+2\left(\delta -x\right)\theta \right)\right)}^{2}\mathrm{dxdy}$$

Rewrite Eq. (4) by discarding the constant terms as follows

(5) $$\left(\theta^{*},\delta^{*}\right)=\arg\max_{\theta ,\delta }\iint \left(P_{0}\left(x,y\right)P_{\pi }\left(2\left(\delta -\mathrm{\theta y}\right)-x,y+2\left(\delta -x\right)\theta \right)\right)\mathrm{dxdy}$$

To find the solution of the above optimization problem, we employ an iterative scheme. At the *k*th iteration, the basic step is given by the following iteration

(6) $$\left({\tilde{\theta }}_{k},{\tilde{\delta }}_{k}\right)=\arg\max_{\theta ,\delta }\int \left(P_{0}\left(x,y\right)P_{\pi }\left(2\left(\delta -\mathrm{\theta y}\right)-x,y+2\left({\tilde{\delta }}_{k-1}-x\right){\tilde{\theta }}_{k‒1}\right)\right)\mathrm{dxdy}$$

The optimizer of Eq. (6) is achieved by using a two-step method which includes performing a composite geometric transformation *T*^*k* − 1^ and then following a linear regression process.

In the *k*th step, *T*^*k* − 1^ is applied to *P*_*π*_ to rotate it with the angle − 2*θ*_*k* − 1_ and then reflect it. Here *T*^*k* − 1^ is defined as follows

$$\begin{array}{ll} T^{k-1} & =\left[\begin{array}{ccc} 1 & 0 & {\tilde{\delta }}_{k-1}\\ 0 & 1 & 0\\ 0 & 0 & 1 \end{array}\right]\;\left[\begin{array}{ccc} -1 & 0 & 0\\ 0 & 1 & 0\\ 0 & 0 & 1 \end{array}\right]\;\left[\begin{array}{ccc} \cos2{\tilde{\theta }}_{k-1} & \sin2{\tilde{\theta }}_{k-1} & 0\\ -\sin2{\tilde{\theta }}_{k-1} & \cos2{\tilde{\theta }}_{k-1} & 0\\ 0 & 0 & 1 \end{array}\right]\;\left[\begin{array}{ccc} 1 & 0 & -{\tilde{\delta }}_{k-1}\\ 0 & 1 & 0\\ 0 & 0 & 1 \end{array}\right]\\ & =\left[\begin{array}{ccc} -\cos2{\tilde{\theta }}_{k-1} & \sin2{\tilde{\theta }}_{k-1} & {\tilde{\delta }}_{k-1}\left(1+\cos2{\tilde{\theta }}_{k-1}\right)\\ \sin2{\tilde{\theta }}_{k-1} & \cos2{\tilde{\theta }}_{k-1} & -2{\tilde{\delta }}_{k-1}\sin2{\tilde{\theta }}_{k-1}\\ 0 & 0 & 1 \end{array}\right] \end{array}$$

$P_{\pi }^{k}$ is obtained by applying *T*^*k* − 1^ to *P*_*π*_. It can be proved that we have

(7) $$P_{\pi }^{k}\left(x,y\right)=P_{\pi }\left(2\left(\delta -\mathrm{\theta y}\right)-x,y+2\left(\delta -x\right)\theta \right)$$

Then we seek to find the solution of the following optimization problem

(8) $$\left({\tilde{\theta }}_{k},{\tilde{\delta }}_{k}\right)=\arg\max_{\theta ,\delta }\iint \left(P_{0}\left(x,y\right)P_{\pi }^{k}\left(2\left(\left(\delta -{\tilde{\delta }}_{k-1}\right)-\left(\theta -{\tilde{\theta }}_{k-1}\right)y\right)+x,y\right)\right)\mathrm{dxdy}$$

Considering the cross-correlation of *P*_0_ and $P_{\pi }^{k}$ along X

(9) $$\left(P_{0}☆P_{\pi }^{k}\right)\left(t\right)=\int P_{0}\left(x,y\right)P_{\pi }^{k}\left(t+x,y\right)\mathrm{dx},$$

Eq. (8) can be rewritten as follows

(10) $$\left({\tilde{\theta }}_{k},{\tilde{\delta }}_{k}\right)=\arg\max_{\theta ,\delta }\int \left(P_{0}☆P_{\pi }^{k}\right)\left(2\left(\left(\delta -{\tilde{\delta }}_{k-1}\right)-\left(\theta -{\tilde{\theta }}_{k-1}\right)y\right)\right)\mathrm{dy}$$

The above equation shows that the optimizer of Eq. (8) can be achieved by computing the cross-correlations of *P*_0_ and $P_{\pi }^{k}$ row by row, and fitting the positions *t*_*m*_(*y*), which are corresponding to the maximum of correlation coefficients, with the following linear model

(11) $$t_{m}\left(y\right)=2\left(\delta -{\tilde{\delta }}_{k-1}\right)-2\left(\theta -{\tilde{\theta }}_{k-1}\right)y$$

The stop criterion of the iterative method is set as both *θ* and *δ* are less than specified tolerance values respectively. *T*^0^ is set to the identity matrix.

## Results

To evaluate the proposed method, we carried out experiments on both synthetic and real in-line XPCI data. MC-based sinogram correction scheme and the proposed method have been implemented using MATLAB (The MathWorks, Inc., Natick, MA, USA). Inverse Radon transform with Ram-Lak filter was taken as CT reconstruction algorithm applied to corrected data. Data processing for this paper was carried out on a Dell workstation system with a 2.4GHz Intel Core i5 processor and 8GB memory.

### Simulation on synthetic data

**Figure 5:**
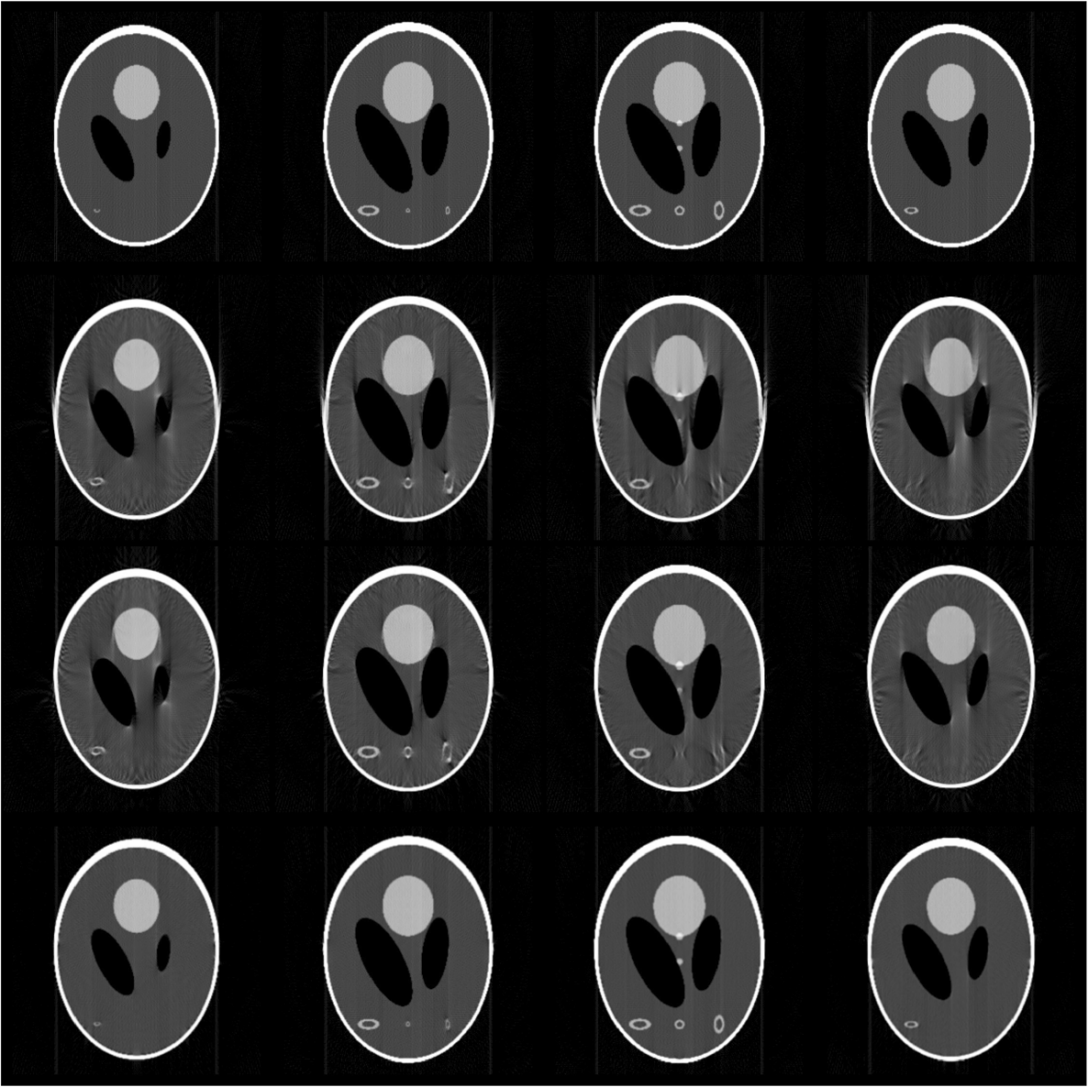
**Four CT slice images of modified 3D Shepp-Logan phantom reconstructed by FBP.** From top to bottom are the results of references, without correction, with MC-based sinogram correction, and with proposed geometric correction method.

For the synthetic case, we used projections of modified 3D Shepp-Long (S-L) phantom in Figure 4 of size 256×256×256 as a test dataset. These projections were obtained by using Radon transform after applying geometric transformation with specified parameters to the 3D S-L phantom. 2 pixels shift along axis X was applied to the rotation axis while flip angle was set to −5 degree. Tilt angle ranged from 0° to 180° and 181 projections were built in the simulation with one projection per degree. Figure 5 shows the results of 4 middle CT slices reconstructed without correction (NC), with MC-based sinogram correction (MC), and with proposed geometric correction method(GC) respectively. By comparing these slice images, we confirm that MC-based sinogram correction scheme reduce the blur effect but cannot remove edge artifacts, and the proposed geometric correction method significantly removes both the blur and edge artifacts at the same time.

For quantitative comparison, we took CT slice images reconstructed from projections without geometric transformation as references, and employed mean Structural SIMilarity (SSIM) index [19] and Mutual Information(MI) [20] to assess the image quality of above results by measuring the similarity between them and the corresponding references. For an image *X* with respect to its reference *Y*, SSIM index is defined as follows

(12) $$\mathrm{SSIM}\left(x,y\right)=\frac{\left(2\mu_{x}\mu_{y}+C_{1}\right)\left(2\sigma_{\mathrm{xy}}+C_{2}\right)}{\left(\mu_{x}^{2}+\mu_{y}^{2}+C_{1}\right)\left(\sigma_{x}^{2}+\sigma_{y}^{2}+C_{2}\right)}$$

where *x* and *y* refer to a local window in the image *X* and *Y* respectively, *μ*_*x*_ (*μ*_*y*_) is the mean while *σ*_*x*_ (*σ*_*y*_) is the standard deviation over the window *x* (*y*), *σ*_*xy*_ is the co-variance between *x* and *y*, *C*_1_ and *C*_2_ are two small positive constants. And the mean of SSIM index (mSSIM) is the average over all local windows. Mutual information is defined as

(13) $$\mathrm{MI}\left(X,Y\right)=H\left(X\right)+H\left(Y\right)-H\left(X,Y\right)$$

where *H*(*X*) and *H*(*Y*) are the Shannon entropy of the image *X* and the reference *Y* respectively, and *H*(*X*, *Y*) is the joint Shannon entropy of the image *X* and its reference *Y*. We calculated mSSIM and MI values for the above slice images and listed the results in Tables 1 and 2 separately. Comparing the results, we can conclude that CT slice images reconstructed from projections pre-processed by the proposed method achieve much higher quality than popular MC-based scheme.

**Table 1:** Comparisons of reconstruction accuracies with mSSIM

**Slice No.**	**NC**	**MC**	**GC**
1	0.57	0.69	0.92
2	0.55	0.76	0.95
3	0.54	0.83	0.96
4	0.51	0.68	0.91

**Table 2:** Comparisons of reconstruction accuracies with MI

**Slice No.**	**NC**	**MC**	**GC**
1	1.52	1.60	2.15
2	1.63	1.83	2.60
3	1.62	1.99	2.73
4	1.50	1.64	2.18

### In-line XPCI data correction experiment

**Figure 6:**
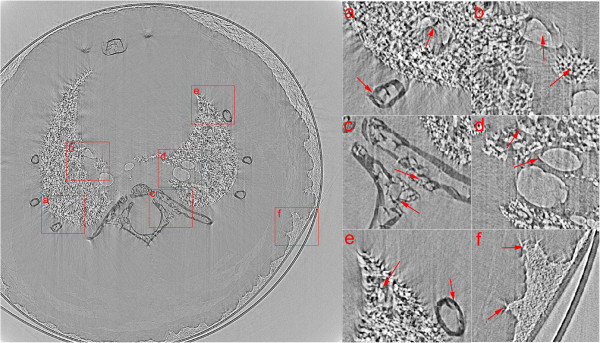
**Reconstructed CT slice from a mouse lung by FBP with MC-based sinogram correction method. (a)**, **(b)**, **(c)**, **(d)**, **(e)** and **(f)** are magnified images of ROIs with corresponding labels in left slice image.

**Figure 7:**
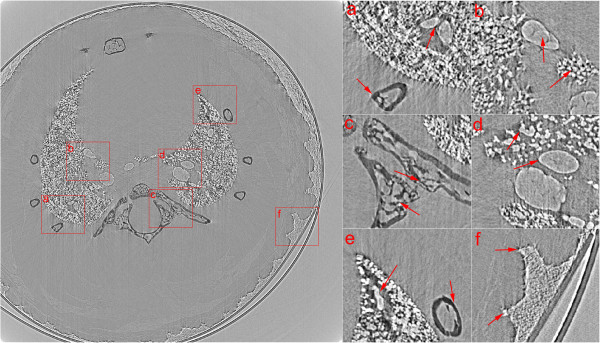
**Reconstructed CT slice from a mouse lung by FBP with the proposed geometric correction method. (a)**, **(b)**, **(c)**, **(d)**, **(e)** and **(f)** are magnified images of ROIs with corresponding labels in left slice image.

Real in-line XPCI data was acquired from a mouse lung at X-ray imaging and biomedical application beamline (BL13W1) of Shanghai Synchrotron Radiation Facility (SSRF). The experiment was approved by the Animal Experiments and Experimental Animal Welfare Committee of Capital Medical University (Beijing, China) and the approval ID is AEEI-2014-049. To prevent from deformation during imaging approach, the mouse lung was fixed in 10% formalin solution, and dried in advance. And then it was put into a small tube, which was rolled by using Kapton film (Dupont, DE, USA), and placed on the rotary stage as illustrated in Figure 1. A Si (111) double-crystal was used to monochromatize the synchrotron X-ray beam. X-rays with photon energy of 18 keV were chosen to provide both good phase and absorption contrast from the mouse lung. An X-ray sensitive CCD detector with high spatial resolution of 9 μm was placed 1.2 m away from the rotary stage along X-ray downstream to record projection images. During the CT data acquisition, the mouse lung was tilted around rotation axis of the stage from 0° to 180°, and 1296 projections were collected totally with exposure time of 80 milliseconds for each one. The rotary stage was coarse adjusted before the imaging process. To improve the computation accuracy, a simple background subtraction approach was employed before correction schemes were taken by deducting mean value of background region from projection images.Figure 6 shows one of CT slices reconstructed by FBP with MC-based sinogram correction method while Figure 7 is with the proposed geometric correction method. Six regions of interest (ROIs) are magnified to better visualize the interior details for comparison. Due to the benefits of correcting misplaced projection pixels by using the proposed method, slice images are reconstructed with much less edge artifacts and blurs. Compared to the slice image in Figure 6, more detailed structural features of alveoli, ribs and bronchus of mouse lung are better displayed in Figure 7. The supporting walls of two adjacent bronchi can be clearly identified as shown in ROI (a). Alveolar and bronchial walls are closed naturally and with less edge artifacts as displayed in ROIs (b), (d) and (e). Structures of ribs are more sharp and without blurred as shown in ROIs (a), (c) and (e). And sharp and detailed image of soft tissue can be observed in ROI (f).

## Conclusions

CT reconstruction of X-ray Phase Contrast Imaging enables to investigate internal microstructure of biological samples with high resolution. Mechanical imperfect or misalignment problem makes the reconstructed slice images blurred and with edge artifacts, and thus destroy the features of microstructures and reduce the spatial resolution of in-line XPCI. To restore the images from collected projections by in-line XPCI, a fast geometric correction method is proposed to determine the geometric transform parameters properly. From the results of numerical experiments on synthetic and real datasets, we have the conclusion that the proposed method can significantly improve the image quality by removing both blurring and edge artifacts at the same time. Geometric correction method utilizes the symmetry of projections, and thus provides a simple and fast scheme to correct misplaced projection data.

## Competing interests

The authors declare that they have no competing interests.

## Authors’ contributions

The work presented here was carried out in collaboration between all authors. WGM and LSQ defined the research theme, and also designed the methods and experiments. WMS and DLN carried out the laboratory experiments, and WGM analyzed the data, interpreted the results and wrote the paper. All authors read and approved the final manuscript.
